# Ultradiluted *Eupatorium perfoliatum* Prevents and Alleviates SARS-CoV-2 Spike Protein-Induced Lung Pathogenesis by Regulating Inflammatory Response and Apoptosis

**DOI:** 10.3390/diseases13020036

**Published:** 2025-01-30

**Authors:** Anirban Roy, Avipsha Sarkar, Asit Kumar Roy, Tanusree Ghorai, Debadatta Nayak, Subhash Kaushik, Satadal Das

**Affiliations:** 1 Virology Laboratory, DAC Regional Research Institute, 50, Rajendra Chatterjee Road, Kolkata 700035, West Bengal, India; avipshasarkar@gmail.com (A.S.); tanusreeghorai11@gmail.com (T.G.); 2 Department of Environmental Science, University of Calcutta, 35, Ballygunge Circular Road, Kolkata 700019, West Bengal, India; asit.roy790@gmail.com; 3 Central Council of Research in Homeopathy, Institutional Area, Janakpuri, New Delhi 110058, India; drdnayak@gmail.com (D.N.); subhashccrh@gmail.com (S.K.); 4 Peerless Hospital and B.K. Roy Research Centre, Kolkata 700096, West Bengal, India

**Keywords:** SARS-CoV-2, S protein, oxidative stress, ROS, cytokines, mitochondria

## Abstract

Background/Objectives: SARS-CoV-2 provokes acute oxidative stress in the lungs via cytokines, inflammatory mediators, and apoptotic factors, which might cause alveolar injury followed by severe respiratory syndrome during COVID-19 infection. The lack of particular antivirals for SARS-CoV-2 has opened novel avenues of complementary and alternative medicine as a potential remedy. The current study explored the mechanistic role of the ultradiluted formulation of Eupatorium (UDE) against SARS-CoV-2 recombinant S protein-mediated oxidative stress and mitochondriopathy. Methods: Cell line and BALB/c mice were used to report that SARS-CoV-2 S protein caused an inflammatory response and subsequent cytokine storm via the NF-κB pathway in the lung along with oxidative damage. Morphological examination was performed using DAPI staining and histology for treated cells and lung tissues of animals, respectively. The molecular mechanism of action of UDE was investigated through qRT-PCR for the genetic expressions of various cytokines, inflammatory, and apoptotic mediators; ELISA, immunofluorescence, immunohistochemistry, and Western blot for the translational expression of the same molecules assayed for genetic expressions; and biochemical assays for various enzymes and ROS. Results: UDE treatment suppressed the inflammatory cell infiltration and tissue-level oxidative stress and safeguarded mitochondrial integrity from free radical-mediated oxidative damage. Additionally, UDE played a direct role in restoring cellular redox homeostasis and reducing the inflammatory response by suppressing NF-κB, IL-1β, IL-18, caspase-1 expression, and ROS formation. Further, a plausible mechanism of action of UDE against S protein-induced damage was proposed. Conclusions: This study described a novel therapeutic approach against S protein-mediated hyperinflammation, apoptosis, and oxidative damage. Hence, UDE may be considered as a prospective alternative to combat life-threatening consequences of SARS-CoV-2 infection.

## 1. Introduction

The occurrence of the novel coronavirus disease 2019 (COVID-19) pandemic caused by SARS-CoV-2 mostly affected the aged and immunocompromised population. Millions of lives were lost because of the virus and posed a new challenge for the medical sciences. The epidemiology, molecular properties, processes, clinical manifestations, and consequences of this viral infection are the subject of thousands of investigations. Still, there is a possibility of new emerging strains that may be deadlier than the ones we already have. Primarily, SARS-CoV-2 causes hyperinflammation and acute respiratory distress syndrome (ARDS) in the lungs via the respiratory tract. ARDS may further result in organ damage because of micro-/macro-thromboembolism, aberrant complement activation, or extended viremia, which may lead to systemic multiple organ dysfunctions [1]. Patients who already suffered from COVID-19 may experience post-acute consequences, and certain symptoms may last for several months. According to the World Health Organization (WHO), long-COVID symptoms could arise from COVID-19-induced hypoxia, decreased cardiac output, myalgia, breathlessness, or chronic fatigue syndrome-like symptoms. The possibility of developing neurodegenerative disorders, cancer, and cardiovascular disorders are a few examples of the long-term negative effects that may occur [2]. According to the literature, these symptoms are a direct result of redox imbalance, excessive free radical formation, mitochondrial degeneration, and decreased oxidative phosphorylation [3].

The literature suggests that SARS-CoV-2 causes COVID-19 pathogenesis through its protruding spike (S) glycoprotein which interacts with the host cell receptor angiotensin-converting enzyme 2 (ACE2). When the ACE2 receptor interacts with the virus via non-covalent interactions, the downstream events take place [4]. Severe symptoms of COVID-19 accompany a process often referred to as a “cytokine storm”, where extensive inflammation takes place because of the uncontrolled release of pro-inflammatory cytokines, growth factors, and interferons; encompasses an expanding tropism of SARS-CoV-2 for vital organs like the heart, kidney, pancreas, etc.; and induces many systemic abnormalities. As a result, a chronic pathological condition becomes established, known as long COVID. During a cytokine storm, prominent rises in serum inflammatory cytokine levels, such as interferon-γ, IL-6, IL-10, IL-8, tumor necrosis factor (TNF) α, blood ferritin level, and soluble IL-2 receptor alpha, are usually present [5].

There are studies where individual structural proteins of SARS-CoV-2 were administered to understand their antigenicity. The S protein induces a variety of inflammatory changes in innate immune cells, e.g., macrophages, monocytes, and neutrophils [6]. Mechanistically, the S protein of SARS-CoV-2 plays a key role in the process of receptor recognition and cell membrane fusion process. It is made up of two subunits, S1 and S2. The S1 subunit contains a receptor-binding domain that recognizes and binds to the host receptor ACE2, which is usually present on epithelial cells of the alveoli, thereby causing viral endocytosis [7]. The immune cells are triggered by viral constituents and by-products of apoptotic as well as necrotic cells, causing the release of inflammatory mediators, and thus hyperinflammation and thrombus in SARS-CoV-2-infected lungs.

The pathogenesis of COVID-19 infections is significantly aided by the development of oxidative stress in alveolar epithelium and endothelium. Though a basal level of reactive oxygen species (ROS) production is required for targeting infection, the excessive production of ROS and low concentration/activity of intrinsic antioxidants creates an imbalance, and consequently oxidative stress, which results in cell and tissue damage [8]. Concurrent inflammation and thrombosis promote the excessive formation of ROS, resulting in a self-sustaining cycle between oxidative stress, cytokine storm, and disease progression [9]. The overproduction of mitochondrial ROS during inflammatory reactions in alveolar cells results in further downstream signaling. Pro-inflammatory cytokines and oxidative stress markers are linked in patients with severe COVID-19, which is supported by the literature [10,11]. In addition, disruptions in ATP synthesis, the electron transport chain, and any malfunctions in the opening of the mitochondrial permeability transition (MPT) pore can stimulate the apoptotic pathway of the mitochondria. Mechanisms like alterations in transmembrane potential and oxidative damage to the mitochondrial DNA, proteins, and membrane phospholipids can also contribute to the above pathway. When mitochondria are damaged, cytopathic hypoxia develops, which causes partial oxygen reduction and ROS formation, resulting in damage to the outer membrane and release of cytochrome C, and eventually cell death and multi-organ failure [12].

Finding a precise treatment for COVID-19 is crucial as the pandemic relapses repeatedly. Moreover, the long-term negative consequences of the existing vaccinations that have been developed to target different SARS-CoV-2 proteins are still unknown [13]. In this context, improvements can be greatly sourced from natural sources. The antiviral actions of various polyphenols from herbal preparations against viral diseases were explored, and interesting outcomes were published; however, the precise mechanisms and pathways of these formulations are still obscure. The ultradiluted formulation of *Eupatorium perfoliatum* (UDE), an extract from a naturally occurring plant that has historically been used as alternative therapeutics for fever and infections, was used in this study. Past investigations demonstrated that the *Eupatorium perfoliatum* extract has antiviral properties against influenza A virus [14]. Previously, researchers from our laboratory demonstrated its role against Dengue virus infection with a precise mechanism of action [15,16]. Herein, we diluted the tincture of *Eupatorium perfoliatum* according to the Pharmacopoeia of India, 2016, and further diluted using one part of the tincture and nine parts of 70% ethanol. The preparation of subsequent dilutions was carried out with 10 μL of the tincture solution and 990 μL of 70% ethanol up to six times. Then, we investigated its action responses in reducing inflammation caused by recombinant S protein both in cultured cells and BALB/c mice. Our previous study on S protein induction in BALB/c mice showed the remodeling of the extracellular matrix and the role of matrix metalloproteinase-9 (MMP-9) during lung fibrosis [17]. We put forth a hypothesis supported by substantial scientific evidence that links oxidative stress to mitochondrial malfunction and explained how it contributes to the exacerbation of the cytokine storm. This study documented the potential role of UDE in mitigating the inflammatory response and ROS-mediated oxidative stress to resist the cytokine storm and subsequent apoptosis during S protein-induced pathogenesis. UDE exhibited significant potential to alleviate mitochondrial dysfunction with reduced toxicity and concurrent apoptosis in the lung caused by S protein. The study from this research can also direct future research and treatment plans with UDE in other respiratory diseases.

## 2. Materials and Methods

### 2.1. Chemicals

SARS-CoV-2 recombinant S protein (Catalogue no. RPO1260LQ) and ELISA kits were procured from Abclonal, MA, USA. Cell culture media, phosphate-buffered saline (PBS), and fetal bovine serum (FBS) were purchased from Invitrogen, Thermo Fisher Scientific (MA, USA). Acetaldehyde-free ethanol and the tincture solution of UDE (Batch no. 0711) was obtained from HAPCO, West Bengal, India. The MTT reagent (Catalogue no. MTT-TC191) and 4,6-diamidino-2-phenylindole (DAPI) reagent (Catalogue no. TC229-5MG), cell culture plasticware, and dimethyl sulfoxide (DMSO) were purchased from HiMedia (Mumbai, India). Fine chemicals, includingprimary and secondary antibodies, the substrate, and the cellular ROS generation kit, were acquired from Santa Cruz Biotechnologies Inc. (TX, USA), Life Technologies (MA, USA), Abclonal (MA, USA), and Sigma-Aldrich Co. (St. Louis, MO, USA).

### 2.2. Reconstitution of SARS-CoV-2 Recombinant and Spike Antigen

SARS-CoV-2 S1 + S2 ECD (S-ECD) recombinant protein labeled with His tag (wild type, produced by cloning in HEK293 cells, which were endotoxin-free and appeared as a band in SDS-PAGE at 35 kDa) was reconstituted in 1 mL sterile distilled water as per the manufacturer’s data sheet and filtered. It was further dissolved in sterile PBS (pH 7.4) for dilution, and finally, a solution was prepared with a concentration of 2.5 µg/mL, which was used in the experiments to mimic the COVID-19 pathogenesis. Endotoxin tests using an E-toxate kit (Sigma-Aldrich, MO, USA), microbial tests, and microscopic examinations were performed to check the presence of any contamination in the prepared tincture.

### 2.3. Cell Culture

The murine macrophage cell line, RAW 264.7, was purchased from National Centre for Cell Science, Pune, India. Cells were preserved in the laboratory in an incubator with a 5% flow of CO_2_ at 37 °C and 95% air. These were grown and maintained in MEM media, containing 10% (by volume) FBS and 1% antibiotic solution. For experiments, cells were seeded (2 × 10^6^ cells/well) into 6-well plates and cultured for 24 h. Serum-free conditions were used to avoid potential drug–protein interactions.

The following groups were included in all cell culture experiments and conducted in triplicates: (i) untreated control, (ii) only S protein treated, (iii) UDE + S protein treated, and (iv) S protein + UDE treated.

### 2.4. In Vitro Cytotoxicity by MTT Assay

The cytotoxicity of S protein and UDE was determined via colorimetric MTT assay. RAW 264.7 cells (1 × 10^4^ cells/well) were cultured in a 96-well plate under optimum conditions. Once the cells were confluent, different wells were treated with varied concentrations of S antigen solution, having a stock concentration of 2.5 µg/mL (0.5–3% of the media volume) for varying periods. In the same way, UDE, having a stock concentration of 100 pg/mL, was applied to the cells (0.5–4% of the media volume) for the standardization of concentration and time of treatment. The experiment was conducted in triplicates, and blank wells contained only the medium and no cells. A graph consisting of dose versus time was prepared to standardize the doses of S protein and UDE.

### 2.5. Wound Healing/Cell Scratch Assay

The impacts of S protein and UDE on the migration or invasiveness of RAW 264.7 cells were examined using this approach [18]. Cells were seeded in 6-well plates and maintained until they attained 85–90% confluency. The growth media was then changed without FBS for 6hbefore administration of either UDE or S protein. Using a sterile p-200 pipette tip, a gap, or “scratch”, was formed on the confluent cellular monolayer. At this point, images of the fields were captured and are referred to as 0 h images. Cells were gently rinsed with PBS. After that, pre- and post-treatment with UDE and S protein were conducted in IC_50_ concentrations, respectively, obtained from the MTT assay, and a serum-free condition was maintained for a further 24 h of incubation. An Olympus inverted microscope with a digital camera was used to study the gap’s closing, and pictures were obtained at 10× magnifications. The size of the wound and its closure were determined using Image J software (V 1.54, NIH, Bethesda, MD, USA), and proliferation potential was calculated as follows:

$$Wound\;healing\;rate=\frac{{Gap\;area}_{0h}-{Gap\;area}_{24h}}{{Gap\;area}_{0h}}\times 100\%$$

### 2.6. DAPI Staining to Analyze Nuclear Morphology

RAW 264.7 cells were inoculated into 6-well plates and incubated for 24 h. Then, the cells were treated with S antigen at its IC_50_ concentration, and the preventive and curative administration of UDE was carried out via incubation for another 6 h. Following the fixation of cells with methanol, they were stained with DAPI (10 µmol/L working solution) and incubated for 30 min in the dark. After washing twice in PBS cells, they were observed under an Andor confocal microscope.

### 2.7. Immunofluorescence

RAW 264.7 cells were grown on glass coverslips. After treatment, they were fixed in 2% paraformaldehyde in PBS. Then, 0.2% (by volume) Triton X-100/PBS and0.1% (*w*/*v*) bovine serum albumin (BSA) were used to treat the cells. Next, the cells were treated with primary antibody for Caspase-3 (1:200 dilution), washed, and then incubated with the secondary antibody that was conjugated with rabbit anti-goat FITC. Coverslips were mounted and magnified 60 times using an Andor confocal microscope. The fluorescence intensity of individual pictures was analyzed using Image J.

### 2.8. Animal Experiment

The study procedures were approved by the Institutional Animal Ethics Committee, (Sanction no. DACRRIH/CPCSEA/IAEC/2021/08). For the experiments, adult BALB/c mice (male and female)with an approximate individual weight of 25–30 g were taken. These mice were kept in the animal house for a week to acclimatize to the conditions of 60 ± 10% relative humidity, 21 ± 2 °C, and 12 h/12 h photoperiod). Before the experiments, animals were randomized by a veterinarian who was independent of study conduct. An acute toxicity study was performed with female mice to know the median lethal dose (LD_50_) of UDE according to the OECD 423 guideline. The dosing of animals and the observation of clinical signs were recorded in a blind manner. There was zero mortality at all tested levels of doses and LD_50_ of UDE upon a single oral administration, per Globally Harmonized Classification System, and the doses belonged to category 5 or were unclassified (LD_50_ cut off is ∞).

The mice were randomly allocated into 4 groups (6 mice/group) as follows: (i) control (oral administration of water only for 3 consecutive days), (ii) treatment with only S-protein intranasally (5 µL from the stock concentration of2.5 µg/mL) for 3 consecutive days, (iii) treatment with UDE orally (30 µL to each animal)once daily for 3 consecutive days followed by S-protein intranasally for 3 consecutive days, and (iv) treatment with S-protein intranasally for 3 consecutive days followed by UDE orally (30 µL to each animal), once daily for 3 consecutive days. *Ad libitum* food and drinking water were provided to them throughout the treatment period except for acute oral toxicity test. A cocktail of ketamine (80–100 mg/kg IP) and xylazine (10–12.5 mg/kg IP) was used for euthanasia for each animal. Lung tissues of each mouse were uniformly inflated using intratracheal instillation of 10% formalin using a 22 g needleat a rate of 200 µL/second until there was a reflux. After 7 days of the last dose administration, all animals were sacrificed; blood by puncturing retro-orbital sinus and lungs were collected. Sera from blood were separated and used to measure the biochemical profile of lungs, as well as antioxidant and oxidative stress markers.

### 2.9. Histology, Immunohistochemistry (IHC), and Immunofluorescence (IF)

For histological examination, a microtome was used to prepare tissue sections of 4–5 µm thickness from paraffin-embedded blocks, which were stained with hematoxylin and eosin. The prepared slides were observed using a light microscope (Olympus, Tokyo, Japan) for histopathological alterations.

The same unstained tissue sections were utilized to verify the localization of MMP-9 and iNOS by IHC. Sections were washed, 1% trypsin in PBS was applied for unmasking antigens, and the endogenous peroxidase activity was blocked by 3% H_2_O_2_. After blocking non-specific sites with 5% BSA in Tris-buffered saline (TBS) (20 mM Tris-HCl, pH 7.4 containing 150 mM NaCl), tissue sections were incubated with primary antibodies (Santa Cruz Biotechnology, Inc., Dallas, TX, USA) diluted in TBS, overnight at 4 °C, and washed thoroughly. Secondary biotinylated antibodies were used to detect the binding of the primary antibodies: goat anti-mouse (Santa Cruz Biotechnology, USA). Next, horseradish peroxidase (HRP)-conjugated avidin and 3,3-diaminobenzidine (DAB)–H_2_O_2_ were used to develop the reaction. Tissue sections were counterstained with Mayer’s hematoxylin and examined using an Olympus microscope. Images were captured with CellSeNS Entry software, V: 1.15 [19,20].

The localization of cytochrome C was checked by IF using the same unstained lung tissue sections. Antigen retrieval from the tissue sections was performed using trypsin (0.05% trypsin, 0.1% CaCl_2_). Followed by blocking with 5% BSA and extensive washing with TBS, the sections were incubated with primary antibodies (Santa Cruz Biotechnology, Inc., Dallas, TX, USA), diluted in TBS, kept overnight at 4 °C, and washed thoroughly. A solution of the FITC-conjugated secondary antibody (Santa Cruz Biotechnology, TX, USA) was then incubated with the sections, and further counterstaining with DAPI was performed. The resultant sections were washed meticulously with PBS, and images were documented using confocal microscope, Andor Technology (Belfast, Northern Ireland, UK) [12].

### 2.10. ELISA of Cytokines

The cytokines and relevant signaling molecules, caspase-1, iNOS, IL-6, and IL-18 were measured using ELISA kits from ABclonal. Standard curves for the molecules were prepared as directed in the literature [21]. Absorbance was measured using an ELISA reader (Bio-Rad Laboratories, Hercules, CA, USA) at 570 nm as per the manufacturer’s instructions.

### 2.11. Mitochondrial ROS Measurement

Mitochondria from mice lung tissues of different groups of animals were isolated using the differential centrifugation method. Fluorescence intensity was measured using a spectrofluorometer (LS3B, Perkin Elmer, MA, USA) with wavelengths of 499 nm and 520 nm as excitation and emission wavelengths, respectively [12]. The data were normalized to normal values, with 100% representing anormal value.

The intracellular ROS level in RAW 264.7 cells was calculated with Life Technologies kit, and micrographs were captured using a confocal microscope. The experiment was based on the uptake of fluorogenic marker 5-(and-6)-carboxy-2′,7′-dichlorodihydrofluoresceindiacetate (carboxy-H_2_DCFDA) in live cells. The fluorescence intensity of each image was evaluated using Image J software.

### 2.12. Succinate Dehydrogenase (SDH) Activity Assay

Based on the reduction of DCIP by phenazine methosulphate (PMS), SDH activity was analyzed spectrophotometrically. Notably, 1 mL of final reaction mixture contained 0.01 mL of diluted enzyme + 0.78 mL of 50 mM Tris-HCl (pH 8.2) + 0.1 mL of 1.5 mM DCIP (10 mM KCN + 0.1 mL of PMS of 1 mg/mL). The absorbance of DCIP was measured at 600 nm after the reaction was initiated with 0.01 mL of 0.5 M sodium succinate (pH 8.0). One unit of enzyme activity corresponded to the quantity of enzyme that reduced 1 μmol of DCIP per min, withan extinction coefficient of 21 mM^−1^cm^−1^ [22].

### 2.13. Activity Assay for NADH Oxidase

NADH oxidase activity was assayed spectrophotometrically in a solution of pH 7.4 consisting of 50 mM Na_2_HPO_4_–KH_2_PO_4_. The reaction began when 0.125 mM NADH was added to the preparation of sub-mitochondrial particles (0.2 mg/mL). Enzyme activity corresponded to the oxidation of NADH, which was measured at 340 nm (ε = 6.22 mM^−1^cm^−1^) [23].

### 2.14. Assay for Lipid Peroxidation

Lipid peroxidation corresponded to the quantity of conjugated diene in the lung epithelial cells which was measured using a spectrophotometer. Lung tissue homogenates were prepared to extract mitochondrial membrane lipid using a chloroform–methanol mixture (2:1, *v*/*v*). The extract was allowed to evaporate to a dry powder in a nitrogen atmosphere at 25 °C, followed by dissolving in n-cyclohexane. Cyclohexane containing lipids were measured at 234 nm, and the results were quantified as micromoles of lipohydroperoxide/mg of protein where ε is 2.52 × 10^4^/M/cm [24]. Total protein estimation was carried out by the Lowry method [25].

### 2.15. Myeloperoxidase Assay

Myeloperoxidase (MPO) enzyme activity was quantified spectrophotometrically using guaiacol as a substrate. The reaction mixture was made with 0.5 mM H_2_O_2_ + 0.4 M guaiacol in phosphate buffer (50 mM, pH 7.4). Then, 1 mL of the reaction mixture was taken, and lung tissue homogenate was added to observe any alterations in the per-minute absorbance of tetraguaiacol at 470 nm using a Multiskan Sky (Thermo Fisher Inc., MA, USA) [26].

### 2.16. RNA Extraction and Semi-qRT-PCR

The lung tissue samples from mice were transferred to vials containing 1 mL of TRIZOL (Ambion, Life Technologies, MA, USA), which were homogenized. The total RNA from lung tissues was extracted according to the kit instructions. By measuring the total RNA at 260/280 and 260/230 nm using a spectrophotometer, the amount of total RNA was quantified.

For each sample of total RNA, reverse transcription was performed as per manuals of the cDNA reverse transcription kits iScript (Bio-Rad Laboratories, CA, USA). The PCR products were separated using 1% agarose gel stained in ethidium bromide and observed under a transilluminator. Relative expressions of mRNAs between different groups of animals, as mentioned in Section 2.8, were compared in quantitative real-time PCR (Bio-Rad CFX96, Singapore). The alterations in mRNA expression were normalized as a fold change with respect to the normal control, and the same was compared to *glyceraldehyde 3-phosphate dehydrogenase* (*GAPDH*). The NCBI blast and SnapGene PCR primer designing platforms were used to create all of the primers for the DNA oligonucleotides [16].

### 2.17. Western Blot

Total proteins were extracted from the tissues with a lysis buffer, followed by the measurement of protein concentrations by the Lowry method [24]. After quantitation under different concentrations and protein separation by polyacrylamide gel electrophoresis, proteins were transferred to polyvinyldiene fluoride membranes through the semi-dry transfer method and blocked for 1 h in 5% BSA. Next, the primary antibodies were dropwise added on the membranes, superoxide dismutase1 (SOD1), iNOS, bcl-2, bax, and caspase-9 incubated overnight at 4 °C. After 3 washes with TBST, HRP-labeled secondary antibodies were added. After 3 TBST rinses, the chemiluminescence solution was added for development using ChemiDoc (Bio-Rad, Laboratories, CA, USA). Individual bands were analyzed for their pixel density using Image J software [12].

### 2.18. Data Interpretation and Statistical Analysis

The data in the graphs are represented as the mean ± standard error. To determine the mean and standard deviation, all data were collected in duplicate and three copies. An analysis of variance was used to see whether there were significant variations between the means. MTT assay, ELISA, and RT-PCR data were analyzed using Sigmaplot. As stated in the text, the Student–Newman–Keuls test (ANOVA) and Student’s *t*-test were used for the statistical analysis. All data analyses were performed using the statistical software Sigmaplot version 10.0. When *p* value < 0.05, a difference was deemed significant.

## 3. Results

### 3.1. UDE Showed Preventive and Therapeutic Potential Against S Protein-Induced Damage on Cultured Cells

To standardize the cytotoxicity dose on cultured cells with UDE, we exposed RAW 264.7 cells with S protein and UDE for up to 24 h, as mentioned in Section 2.4 and the data presented in Supplementary Figure S1A,B. When more than 1% concentration of the S protein was applied in the media (the final concentration was equivalent to 25 ng/mL), cell viability decreased by 50% after 6 h. In contrast, when UDE was applied to the cells up to 4% (the final concentration was equivalent to 0.04 pg/mL) concentration of the total media volume, we did not observe any significant loss in cell viability. It showed that 72% of cells survived after 24 h. The death of cells in higher concentrations was probably because of the accumulation of some metabolic end products along with some debris in the wells due to the exhaustion of media. So, 1% up to 6 h and 4% up to 24 h were the standardized IC_50_ doses and durations of S protein and UDE, respectively, for further experiments.

**Figure 1:**
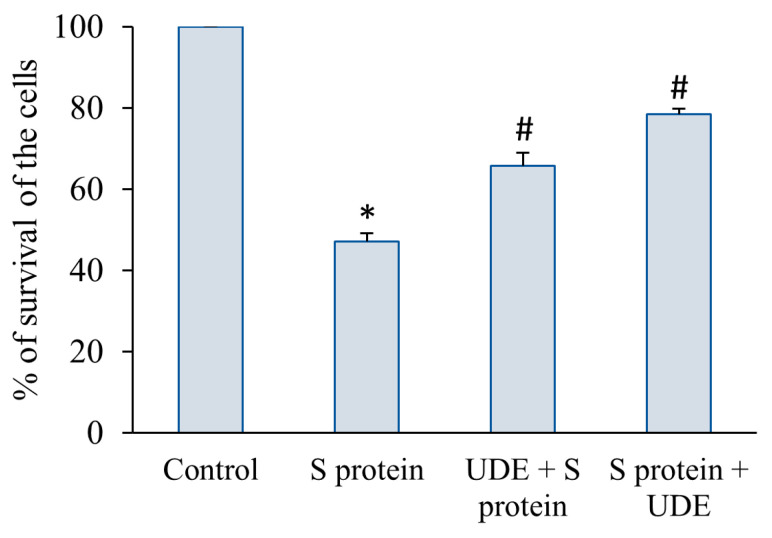
**Cell viability on preventive and therapeutic treatment with UDE against S protein-induced damage.** Cell viability was evaluated with only S protein, 12 h pre-treatment with UDE followed by S protein, and 6 h treatment with S protein followed by UDE. The graphs represent the mean± standard error values of each group. The error bars display standard error of the mean.* indicates a significant difference from the control at *p* < 0.05, and # indicates significant difference from the S protein-treated group at *p* < 0.05.

To assess the preventive and therapeutic efficacy of UDE against the damaging effect of S protein on the viability of RAW 264.7 cells, an MTT assay was performed. For treatment with an IC_50_ concentration of S protein on RAW 264.7 cells for 6 h, viability decreased significantly by almost 52%. The 12 h pre-treatment with UDE increased the viability by 37% which was statistically significant. When UDE was applied after treatment with S protein for 6 h, the viability significantly increased, by approximately 65%, as presented in Figure 1.

**Figure 2:**
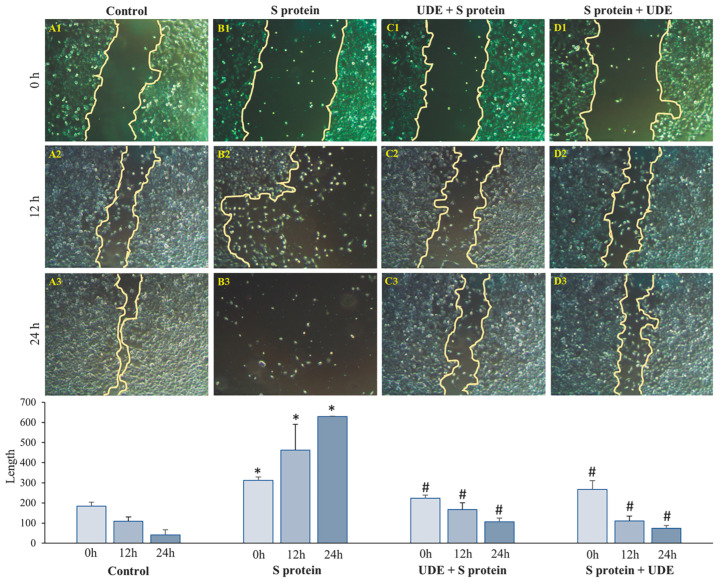
**Scratch assay:** The cell scratch experiment revealed that the S protein dramatically reduced cell invasion into the wound location in 24 h (**B1**–**B3**), while UDE treatment, both preventative (**C1**–**C3**) and curative (**D1**–**D3**), rectified the condition. Images from an inverted light microscope (scale bar: 100 µm) are shown. The bar demonstrated the arbitrary quantitation of the percentage of wound closure or gap filling as the effect of UDE. Graphs represent the mean of three independent experiments (mean ± standard deviation). Error bars in the graphs correspond to the standard error of the mean. * indicates significantly different values with respect to control at *p* < 0.05, and # indicates significantly different values from S protein-administered group at *p* < 0.05.

The wound-healing potential of UDE was assessed by performing a wound healing/scratch assay on RAW 264.7 cell lines, as depicted in Figure 2. After being confluent in 6-well plates, the S protein was applied for 6 h. In two other wells, UDE was applied 12 h before S protein application and 6 h after the administration of the S protein. After making a scratch, all wells were observed for the next 24 h for the closure of the gap. The S protein decreased the proliferation of cells into the margin of the scratch, resulting in no closure of the gap and more damage to the cells (Figure 2(B1–B3)) in comparison to the untreated set of wells (Figure 2(A1–A3)), where the width of the scratch was effectively bridged. The percentage of the gap was found to decrease in control cells, from 100% to 77%, whereas S protein-treated cells lost their proliferation potential, and the gap increased to more than 100% due to the excessive death of the cells. The preventive (Figure 2(C1–C3)) and therapeutic administration (Figure 2(D1–D3)) of UDE filled the gap by 52% and 72%, respectively, in 24 h, when compared to 0 h, which are statistically significant.

### 3.2. UDE Maintained Nuclear Integrity Against S Protein-Mediated Damageand Further Apoptosis

**Figure 3:**
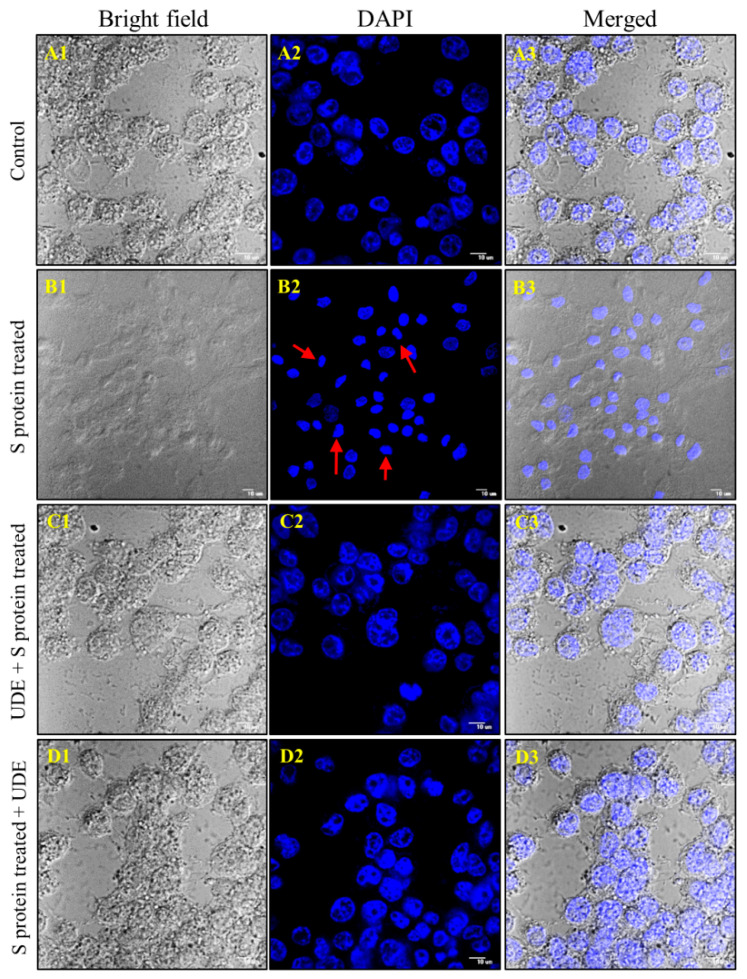
**Confocal microscopy of DAPI-stained RAW 264.7 cells.** RAW 264.7 cells were exposed to the S protein for 6 h with the preventive and therapeutic application of UDE for before and after 12 h each. Nuclear morphological changes and damage, such as chromatin condensation and marginalization (red arrows), are evident in cells exposed to S protein (**B2**) as compared to control (**A2**), whereas no marked nuclear alterations were visible in cells exposed to either preventive (**C2**) or therapeutic administration of UDE (**D2**). (**A1**–**D1**) represented the bright field pictures, and (**A3**–**D3**) represented the merged pictures. Images are representative of three independent experiments. Magnification: 60×; scale bar: 10 µm.

DAPI-stained pictures in the middle panel A2 to D2 of Figure 3 revealed a damaged nucleus with gross distortion, shrinkage of the nuclear membrane, and chromosomal aberration, which indicates cell damage and apoptosis. UDE treatment, both prophylactic and therapeutic, reversed the nuclear damage, and the micrographs C2 and D2 of Figure 3 appeared similar to control A2.

When RAW 264.7 cells were stained with the EtBr-AO cocktail, the various apoptosis stages could be detected, as shown in Supplementary Figure S2.

**Figure 4:**
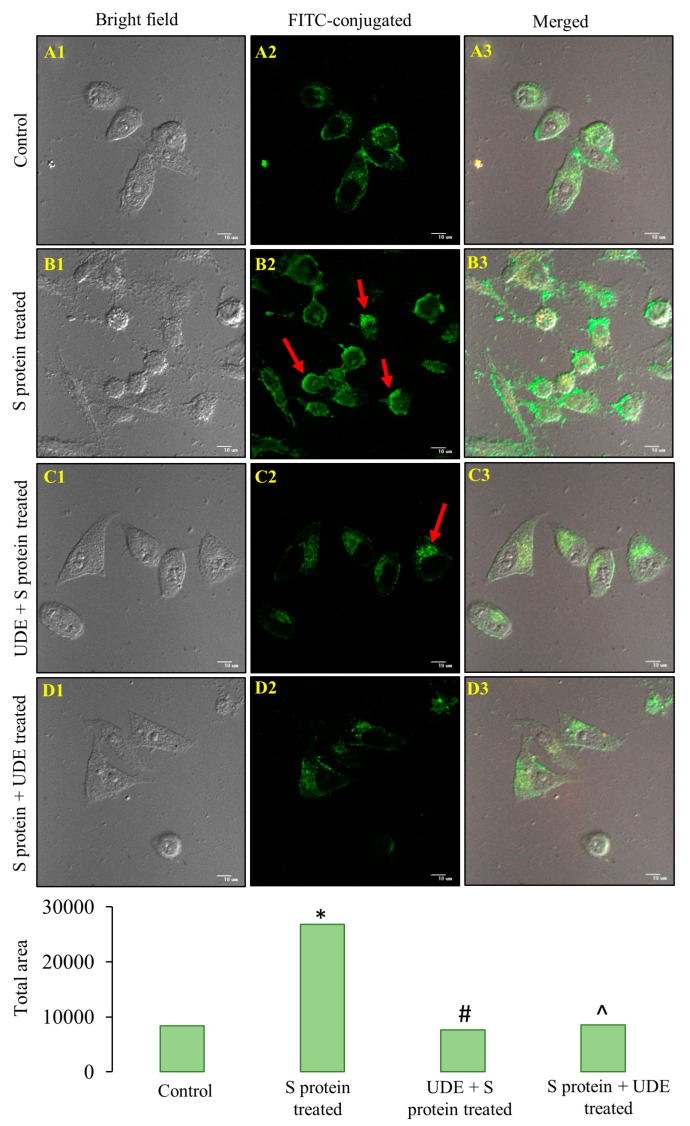
**RAW 264.7 cells stained with caspase-3 following treatment with S protein and UDE.** The bright field images are shown in the left panel (**A1**–**D1**), FITC-conjugated caspase-3 localization in cell cytoplasm are shown in the middle panel ((**A2**–**D2**), red arrows), and the merged bright field and FITC-conjugated caspase-3 images are shown in the right panel (**A3**–**D3**). For the treatment with UDE, the expressions of caspase-3 and morphological deformities were dramatically reduced. Magnification: 60×; scale bar: 10 µm. In the bar graph for fluorescence intensity quantification using Image J, * indicates significant difference from control at *p* < 0.05, # specifies significantly different values from S protein-treated group at *p* < 0.05 in prophylactically UDE-treated group, and ^ indicates significant difference from S protein-treated group at *p* < 0.05 in therapeutically UDE-treated group.

We carried out the immunofluorescence of caspase-3, one of the effector proteins of the apoptotic pathway, which is shown in Figure 4, to further examine the apoptosis of cells after treatment with the S protein. After S protein treatment for 6 h, we found that caspase-3 significantly increased in RAW 264.7 cells, as depicted in Figure 4(B2,B3). The results supported the findings of Supplementary Figure S2. Pre-treated cells with UDE for 12 h significantly reduced cellular damage and caspase-3 production. When UDE was administered therapeutically for the next 12 h (Figure 4(D2)), caspase-3 was significantly downregulated and nearly on par with the control (Figure 4(A2)).

### 3.3. Effect of UDE on ROS Generation in Cells

**Figure 5:**
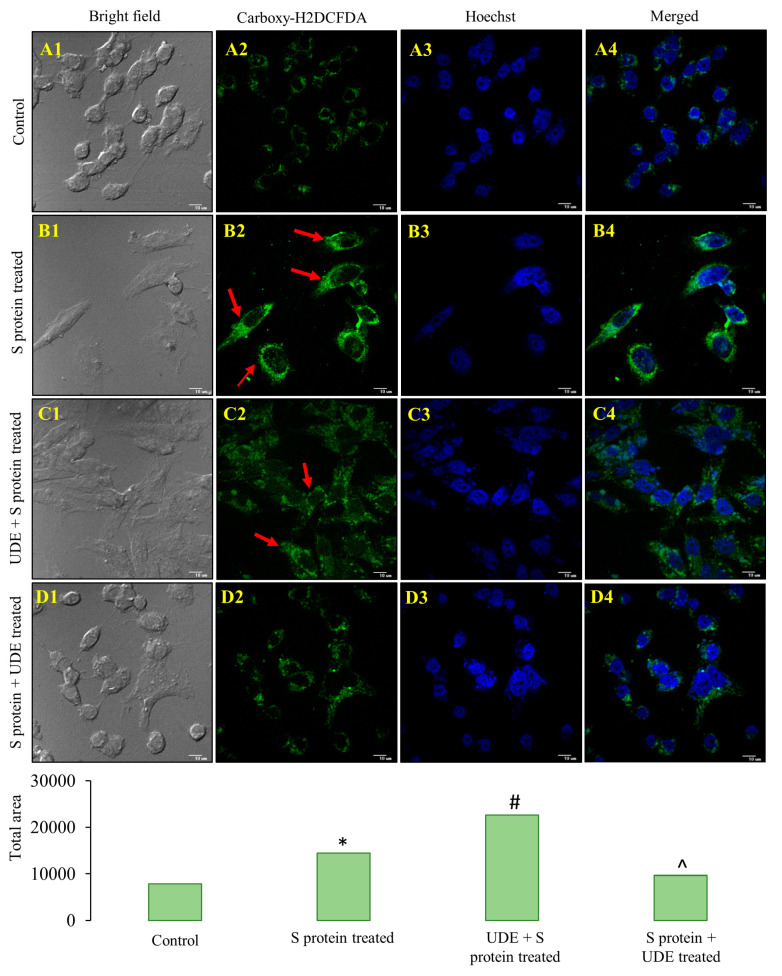
**Detection of ROS production in RAW 264.7 cells with UDE treatment.** Intracellular ROS productions were detected by carboxy-H_2_DCFDA (**A2**–**D2**). Counterstaining with Hoechst was depicted in (**A3**–**D3**). Merged pictures were presented in (**A4**–**D4**). Red arrows in (**B2**,**C2**) indicate generation of ROS, which were statistically significant when fluorescence intensities were compared with control (**A1**–**D1**), measured using Image J software. In the bar graph for fluorescence intensity quantification, * indicates statistically significant from control at *p* < 0.05, # indicates significance with respect to S protein-treated group at *p* < 0.05 in prophylactically UDE-treated group, and ^ denotes significance with respect to S protein-treated group at *p* < 0.05 in therapeutically UDE-treated group.

S protein-treated RAW 264.7 cells induced higher ROS production compared to the control set of cells, the while preventive and therapeutic application of UDE significantly lowered ROS production, as depicted in confocal micrographs A2 to D2 in Figure 5. Counterstaining with the Hoechst stain showed condensed chromatin and aberration of nuclei in S protein-treated cells for 6 h (Figure 5(B3)) which was in the same line of finding with Figure 3(B2). UDE pre-treatment and post-treatment for 12 h each retained nuclear integrity as control cells compared to the S protein-administered cells, along with a significant reduction in ROS generation in the cells (Figure 5(C2,D2)).

### 3.4. Estimation of Cytokine Expression Pattern in Sera of Mice

**Figure 6:**
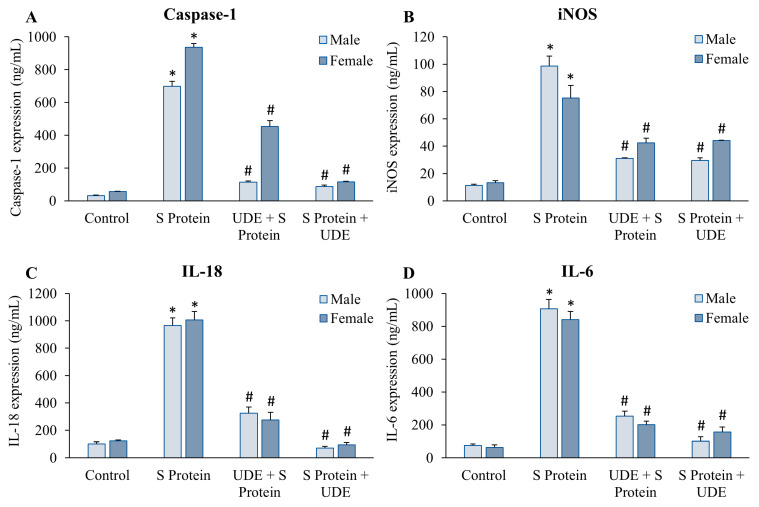
**Cytokines were measured by ELISA in mouse sera after treatment with S protein and prophylactic and therapeutic administration of UDE.** Sera from different groups of mice were subjected to analysis for (**A**) Caspase-1, (**B**) iNOS, (**C**) IL-18, and (**D**) IL-6. Results were expressed as ng/mL total protein. The error bars represent standard error of the mean. * indicates statistically significant with respect to control at *p* < 0.05, and # denotes significant difference from S protein-treated group at *p* < 0.05.

ELISA tests for selected proteins in male and female mice from different groups were performed separately and plotted against the standard. The test samples were diluted to 1:100. The circulating cytokine levels in the blood differed significantly between the control and S protein-treated sets, as presented in Figure 6 and Table 1. In addition, UDE treatment exhibited robust control in the pro-inflammatory axis of cytokines involving caspase-1, iNOS, IL-18, and IL-6.

Upon the intranasal administration of S protein, caspase-1 expression was approximately augmented by 22- and 17-fold in male and female animals, respectively, compared to the control. The prophylactic administration of UDE controlled the level up to 6- and 2-fold in male and female animals, respectively. The therapeutic administration of UDE reduced it more significantly, by 8-fold in both male and female mice, in comparison to S protein-treated mice, as shown in Figure 6A and Table 1. iNOS followed a similar trend where it was upregulated by 9-fold in males and 5-fold in females due to S protein administration. UDE treatment, both prophylactic and therapeutic, reduced levels by approximately 3- and 1.7-fold in male and female animals, respectively (Figure 6B). IL-18 expression in the sera of mice was significantly high due to S protein administration. The therapeutic addition of UDE lowered it very significantly compared to preventive treatment, by approximately 13-fold and 10-fold in males and females, respectively (Figure 6C). IL-6 expression was very high in only S protein-treated animals, whereas its level was significantly downregulated in both male and female animals when they were treated with UDE (Figure 6D).

### 3.5. Analysis of Different Genes from Mice Lung Tissue Using qRT-PCR

The expression of various genes in the mice lung tissues challenged with S protein and treated with UDE were analyzed using qRT-PCR. Based on the physiological functions, the genes were selected into four groups: (1) cytokines and enzymes regulating inflammation (*IL-6*, *IL-10*, *TNFα*, *MMP-9*, *iNOS*), (2) apoptotic regulators (*caspase-3*, *caspase-9*, *bax*, *bcl2*); (3) downstream genes following inflammasome activation (*caspase-1*, *IL-1β*, *IL-18*); and (4) transcription factor (*NFκB*). The expressions of all the selected genes were markedly altered on S protein administration; therefore, the prophylactic and therapeutic treatment of UDE regulated the expressions, and the cross-talk between them was elucidated.

**Figure 7:**
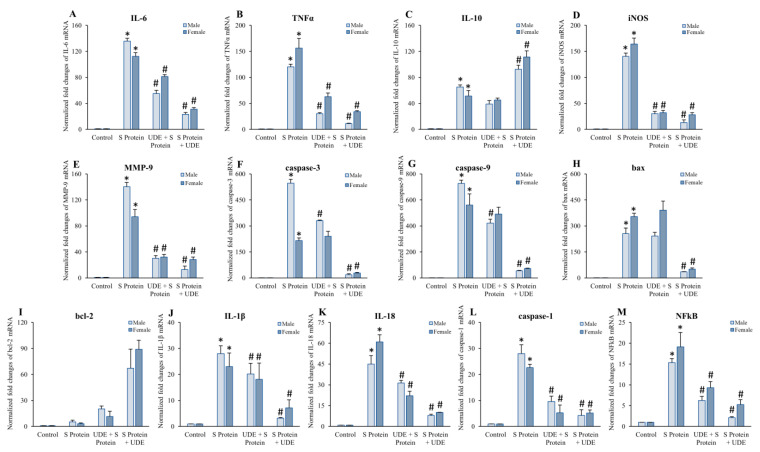
**qRT-PCR of differentially expressed genes in the mice lung of 4 different groups:** qRT-PCR was performed using SYBR Green Mastermix (Biorad) in the CFX96 real-time PCR system. Target gene expression levels were normalized against that of *glyceraldehyde-3-phosphate dehydrogenase* (*GAPDH*). The results revealed that S protein induced significant upregulation of genes related to inflammation, i.e., *IL-6* (**A**), *TNFα* (**B**), *IL-10* (**C**), *iNOS* (**D**), and *MMP-9* (**E**); upregulation of pro-apoptotic genes *caspase-3* (**F**), *caspase-9* (**G**), *bax* (**H**); downregulation of anti-apoptotic gene *bcl-2* (**I**); and upregulation of downstream genes after inflammasome activation *IL-1β* (**J**), *IL-18* (**K**), *caspase-1* (**L**), and *NFκB* (**M**) in both male and female mice. Prophylactic and curative treatment of UDE downregulated the expression of pro-inflammatory genes, pro-apoptotic genes, and genes downstream theinflammasome activation, thereby arresting the disease severity and progression. Bars represent means + SEM for the number of animals. * indicates statistically significant with respect to control at *p* < 0.05, and # denotes significant difference from S protein-treated group at *p* < 0.05.

The expressions of *IL-6* and *TNFα* were found to be elevated by approximately 135-fold and 182-fold in males and 112-fold and 156-fold in females, respectively, in the S protein-treated animals when compared with the control, normalized with the *glyceraldehyde-3-phosphate dehydrogenase* (*GAPDH)* gene expression. Interestingly, the therapeutic administration of UDE reduced *IL-6* expression significantly in both male and female animals. The preventive application of UDE was note worthy in male animals but not so prominent in female animals (Figure 7A). A significant reduction in the *TNFα* mRNA expression was also observed in the therapeutic UDE treatment group, both in males and females (Figure 7B). However, the fold reduction in male animals was more prominent. The RT-PCR of *IL-10* mRNA showed an upregulation of 65- and 51-fold in male and female mice, respectively, in S protein-administered groups, compared to the control. The therapeutic administration of UDE further upregulated the *IL-10* transcription up to 92- and 111-fold in males and females, respectively, in comparison to the control, which is approximately a 2.5-fold difference in comparison to the S protein-treated group (Figure 7C).

The RT-PCR of *iNOS* mRNA also showed a significant upregulation of 140- and 164-fold in males and females, respectively, in the group treated with S protein when compared to the control. Both the preventive and therapeutic administration of UDE significantly downregulated it in male and female mice (Figure 7D). Forthis connection, we also examined the transcriptional expression of *MMP-9* where S protein upregulated the gene nu approximately 140-fold and 164-fold in male and females, respectively. These data also corroborated our previous results [17]. The preventive and curative treatment of UDE significantly downregulated *MMP-9* mRNA expression both in male and female animals (Figure 7E).

When we checked the gene regulation of apoptotic regulators *caspase-3* and *caspase-9*, it was found that both were significantly upregulated in response to the S protein compared to the control, by approximately 546- and 726-fold in males and 215- and 560-fold in female animals, respectively. The curative administration of UDE was able to bring it down significantly, very near to the control, but prophylactic UDE was not very effective in reducing apoptosis (Figure 7F,G). In addition, the expressions of *bax* and *bcl-2* were also modulated towards apoptosis in response to the S protein. *bax* mRNA was found upregulated by 256- and 340-fold in males and females, respectively. The therapeutic administration of UDE significantly downregulated it by approximately 7.5- and 7-fold in male and female animals, respectively, with respect to S protein-induced upregulation. In contrast, the anti-apoptotic *bcl-2* gene was found markedly upregulated by approximately 13.4- and 29.6-fold in males and females, respectively, in response to the therapeutic UDE administration (Figure 7H,I).

While considering the downstream factors following inflammasome activation, the expressions of *IL-1β*, *IL-18*, *caspase-1*, and *NFκB* were checked and found significantly higher in the S protein-treated group compared to the control animals (Figure 7J–M). A significant reduction was found in all the genes in therapeutic UDE-treated groups. However, prophylactic UDE-treated groups did not show a marked consistent reduction. S protein induced upregulation by approximately 28- and 23-fold in males and females, respectively, for *IL*-*1β* mRNA; 45- and 61-fold in males and females, respectively, for *IL*-*18* mRNA; 28- and 22-fold in males and females, respectively, for *caspase-1* mRNA; and 15- and 19-fold in male and female mice, respectively, for *NFκB* mRNA. The therapeutic administration of UDE reduced the expressions in all cases, especially in male mice. The fold changes are depicted in Figure 7. To clarify whether these changes in gene expression would be reflected in the translational level, we performed Western blot, IHC, and IF of the same and related proteins.

### 3.6. Biochemical Investigations of Mice Lung Tissue Extracts

The intranasal administration of S protein in mice significantly increased the lipid peroxidation level in all animals, as shown in Table 2. The preventive and curative administration of UDE lowered the conjugated diene significantly, which were equivalent to 41% and 74%, respectively, in males and 69% and 78%, respectively, for the S protein-treated group, and were statistically significant.

The anti-inflammatory role of UDE was evaluated by its capacity to prevent MPO activity, which is a known marker for inflammation. Compared to the S protein-treated group, MPO activity was significantly lowered in the therapeutically treated groups, which was very close to the control values, equivalent to 74% in males and 80% in females. In contrast, preventive treatment with UDE could not reduce MPO activity when compared to the therapeutically treated group.

Intercellular antioxidant level is directly proportional to the amount of total thiol groups. Treatment with S protein significantly reduced these groups, by approximately 35% and 58% in male and female mice, respectively, when compared to the control. The total thiol groups were restored by preventive and therapeutic UDE administration in both male and female animals, which were comparable to the control animals, as presented in Table 2. These results suggested that the treatment of UDE significantly reduced inflammation and oxidative load caused by S protein challenge in mice.

S protein-treated mice lungs showed approximately 71% and 81% decreased activity of SDH with a 79% and 83% increase in NADH oxidase in males and females, respectively, compared to their normal counterparts, which were statistically significant. However, these levels appeared comparable to the control group when the mice were treated with UDE either prophylactically or therapeutically to heal lung damage, as shown in Table 2.

Mitochondrial ROS production significantly increased in the lungs of mice after treatment with S protein in both sexes of animals. The amount of ROS produced in mitochondria was directly proportional to the intensity of the fluorescence H_2_DCFDA produced upon oxidation to H_2_DCF. The relative fluorescence intensity increased in S protein-treated male and female mice by 83% and 86%, respectively, for the baseline values of control group animals. Both the preventive and curative administration of UDE significantly lowered ROS generation in all the animals. However, curative administration of UDE reduced the values more significantly (66% in males and 75% in females), close to control values, which also corresponded to the finding from cell culture experiments, as depicted in Figure 5.

### 3.7. Induction of Protein Expression in Mouse Lungs After S Protein Challenge

**Figure 8:**
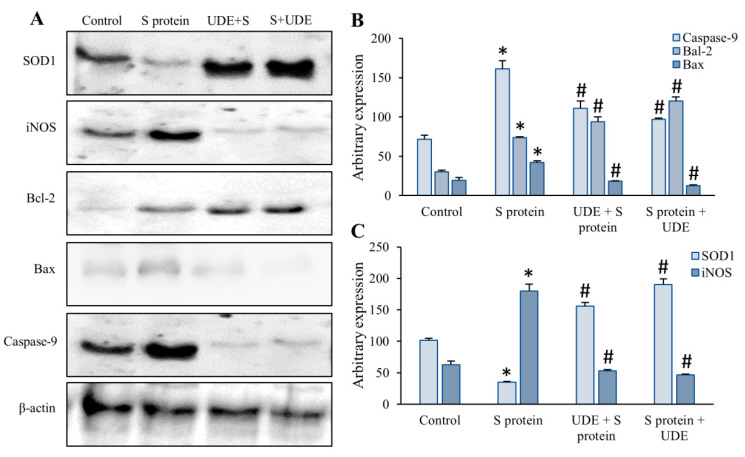
**Western Blots of SOD1, iNOS, Bcl-2, Bax, and caspase-9** (**A**) Treatment with S protein significantly altered the expressions of SOD1, iNOS, Bcl-2, Bax, and caspase-9, and UDE treatment restored the protein expressions close to control values. β-actin blot was given as a loading control. (**B**) Pro-apoptotic caspase-9, Bax, and anti-apoptotic Bcl-2 were plotted for different bands that came in the blots. (**C**) The graphs represent the expression of antioxidant enzyme SOD1 that was plotted against inflammatory iNOS. Error bars represent means ± SEM. * denotes a statistically significant difference from control at *p* < 0.05, and # denotes a statistically significant difference from S protein-administered group at *p* < 0.05.

To verify the translational expression of a few proteins, Western blotting was performed with lung tissue extracts from mice of different groups (Figure 8). Of the genes identified as key players in the proposed pathway, we selected those proteins that play a major role in apoptotic and redox homeostasis, such as SOD1, iNOS, Bcl-2, Bax, and caspase-9. Our results showed the dysregulation of all selected proteins in response to the S protein. Pre- and post-treatment with UDE controlled the expressions given in Table 3. The expression of SOD1 significantly increased with both the pre- and post-treatment of UDE over the S protein, which signifies the restoration of cellular antioxidant pool and decrease in ROS. The expression of iNOS was significantly decreased with the prophylactic and therapeutic administration of UDE, which was approximately 3-fold upregulated in response to the S protein. While considering the intrinsic pathway of apoptosis, the S protein induced a 2.2-fold increase in both pro-apoptotic proteins, Bax and caspase-9. The therapeutic treatment of UDE potentially downregulated it to approximately 3.3- and 1.6-fold, respectively, when compared to S protein-administered lungs. In addition, the translational expression of anti-apoptotic Bcl-2 also increased with the prophylactic and therapeutic treatment of UDE, though they were not significant.

### 3.8. Histology Analysis of Mice Lung Tissues After Treatment

**Figure 9:**
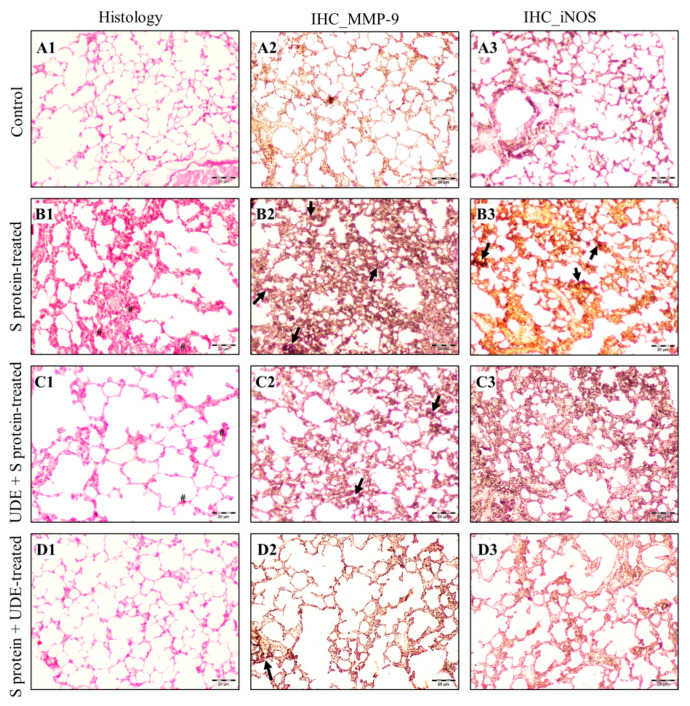
**Representative photomicrography of mice lung sections of BALB/c mice from the groups**. H&E-stained sections (**A1**–**D1**) showed inflammation-associated changes to bronchiolar and alveolar architecture along with other pathological changes: (**A1**) section of the untreated lung (control); (**B1**) section of the lung of a mouse in the S-protein treated group, where mice (*n* = 5) were administered S protein intranasally for consecutive 6 days; (**C1**) section of the lung of a mouse from the preventive group (treated with UDE before S protein administration); and (**D1**) section of the lung of a mouse from the therapeutic group (treated with S protein followed by UDE). Inflammation in the lung and accumulation of inflammatory exudate are denoted by black arrowheads. Alveolar macrophages are denoted by ‘#’. Immunohistochemistry for MMP-9 expression (**A2**–**D2**) and iNOS (**A3**–**D3**) were depicted in the mice lung tissue sections. Black arrows indicate the immunoexpression of proteins. Magnification: 10×. Scale bars represent 20 μm.

The analysis was performed from the histological examination of the conducting airway structure (bronchioles) and gas exchange area (respiratory bronchioles, alveolar ducts, and sacs) in each group (Figure 9 and Table 4). Lung sections from the control, S protein-treated, preventive, and therapeutic UDE-treated groups revealed significant differences concerning overall lung architecture and other pathologies. In micrograph A of Figure 9, the histological findings of alveoli and interalveolar septa of the lungs of mice appeared normal with usual airspaces and thin alveolar septa when no treatment was given. Intranasal S protein administration for 3 consecutive days caused pathogenicity (B1) in the mice lung, where moderate inflammatory exudates accumulated in the interalveolar septa (‘#’ in Figure 9(B1,C1)) with inflammatory cell infiltration in the tissue spaces, and significantly higher alveolar macrophages (probably iron-laden) and congestion in airway structures were seen. There were non-uniform dilatations of alveoli, almost twice compared to the control, due to the coalescence of the adjacent ruptured alveoli, and decreased airspace areas due to the significant thickening of alveolar septa that were seen in many fields. In contrast, UDE pre-treated lung tissue in micrograph C1 showed minimal inflammatory infiltrate and exudates in comparison to S protein-treated lungs. Very few alveolar macrophages were seen in the pulmonary airspaces and in alveolar walls, and dilatations of the alveoli significantly decreased. Alveolar diameters were the same as seen in S protein-treated tissues and were more diverse. In contrast, the lung with therapeutic treatment with UDE (D1) showed normal histology, comparable to the control, without any significant pathological changes. There was a decrease in congestion in the alveoli and respiratory bronchioles. The presence of inflammatory exudates was extremely mild, and alveolar macrophage number was normal. Alveolar diameter also became comparable to the control and more uniform. The extent of inflammatory cell infiltration and airspace areas in the therapeutic UDE-treated tissues were significantly lower than that in the S protein-treated tissues. Both the preventive and curative treatments of the lung with UDE resulted in conditions that were significantly protected and cured, with very few pathological changes.

### 3.9. Immunofluorescence and Immunohistochemistry of Proteins in Lung Tissues

Only homogenized lung sample data were presented from RT-PCR and Western blot investigations. As a result, we attempted to identify the cell type(s) or structure(s) in the lung sections that may be responsible for the manifestation of the S protein-induced expressions of MMP-9, iNOS, and cytochrome C, which are depicted in Figure 8 and Figure 9.

To investigate the accumulation of MMP-9 and iNOS in the mice lung tissues, we examined histological sections prepared from paraffin-embedded blocks of lung tissues from all four groups of BALB/c mice using polyclonal antibodies (Figure 9(A2–D2) for MMP-9 and A3 to D3 for iNOS). MMP-9 immunoexpression was more or less detected as intense brown (arrowheads in the Figure 9) in all the groups of mice, but their localization intensity varied considerably in the following different groups: (A) control, (B) S protein-treated, (C) UDE pre-treated and S protein-treated, and (D) S protein followed by UDE-treated. In S protein-treated samples, MMP-9 was more densely localized in the regions of interalveolar septa (arrowheads in Figure 9(B2)), which were solidified for the accumulation of exudates in comparison to the control tissue sections. On the contrary, the IHC examination of lung tissue samples pre-treated or post-treated with UDE along with the S protein showed very scanty brown, implying a limited localization of MMP-9 (Figure 9(C2,D2)). A similar trend of immunoexpression of iNOS was found in MMP-9, i.e., more expressions in S protein-treated tissues and considerably fewer expressions in UDE pre-treated or post-treated tissues. Interestingly, therapeutically administered UDE showed significantly less occurrences of both MMP-9 and iNOS.

**Figure 10:**
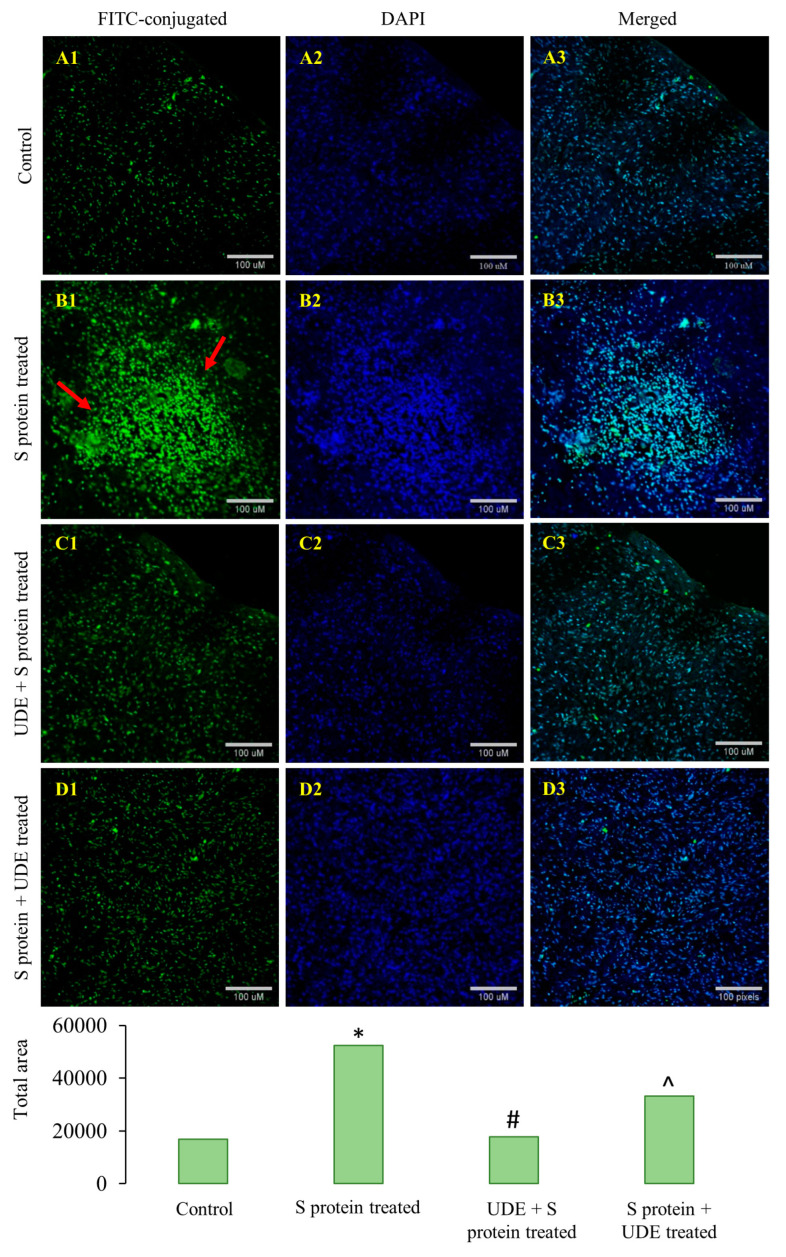
**Confocal microscopy images of mice lung sections immunofluorescently labeled for cytochrome C.** Images represent control, S protein-treated, UDE + S protein-treated, and S protein + UDE-treated groups. (**A1**–**D1**) represents immunostaining of cytochrome C, (**A2**–**D2**) represents DAPI as counterstaining, and (**A3**–**D3**) micrographs are the merged pictures, showing cytochrome C accumulation in the tissue spaces. Red arrows in (**B1**) showed significantly high fluorescence indication for cytochrome C, which was detected by FITC-conjugated secondary antibody. There was a significantly high expression of cytochrome C in S protein-treated mice lungs compared to the control group. Scale bars: 10 µM. In the bar graph for fluorescence intensity quantification, * denotes a statistically significant difference from the control group at *p* < 0.05, # denotes significant difference from S protein-administered group at *p* < 0.05 in prophylactically UDE-treated group, and ^ denotes significant difference from S protein-administered group at *p* < 0.05 in therapeutically UDE-treated group.

The expression and accumulation of cytochrome C were examined in the mice lung tissues by confocal microscopy, as presented in Figure 10. A significantly higher expression of cytochrome C appeared in S protein-administered tissues. Mice lung parenchyma, including the alveoli, alveolar ducts, and respiratory bronchioles, were analyzed, which under went positive staining with cytochrome C antibody (Figure 10(A1–D1)). Nuclei were stained with 4,6-diamidino-2-phenylindole (DAPI; blue) depicted in the middle panel (Figure 10(A2–D2)). Both the prophylactic and therapeutic administration of UDE reduced cytochrome C expression compared to the control group, and the data corroborated the finding obtained from the qRT-PCR of cytochrome C.

## 4. Discussion

The attempt to reduce the impact of the COVID-19 disease burden is an important concern for the research community and global healthcare. To gain insights into the mechanisms of disease progression, virus evasion strategies, and host responses and to create efficient antiviral therapeutics, animal models mimicking viral infection and subsequent pathogenesis are extremely important. Several critical lung disorders, including COVID-19 and ARDS, may lead to pulmonary fibrosis, destroy alveolar shapes, and impair lung function. But, before proceeding to animal models, cell-based models were utilized, and the results were further extrapolated to BALB/c mice for validation [29]. To mimic the general physiological and pathological processes, we used mouse macrophage cell line RAW 264.7 and administered S protein in vitro to assess the preventive and therapeutic effects of UDE. This cell line is a well-known in vitro disease model for investigating the bioactivity of therapeutic agents and their efficacy, which are indicative of the potential response in human cells. Wang et al., 2022, described the role of alveolar macrophages as first-line defenders that scavenge the inhaled pathogen or xenobiotics to maintain lung homeostasis [30]. Studies demonstrated that viruses not only infect alveolar macrophages but also replicate in the lower respiratory tract, causing cytotoxicity, overproduction of pro-inflammatory cytokines and interferons, and finally, fatal pneumonia [31]. From the histological pictures, we showed the infiltration of inflammatory cells and macrophages in the lung of S protein-treated group of mice.

ROS and reactive nitrogen species (RNS) in cells and tissues are a double-edged sword because they can affect physiological function both favorably and unfavorably. Beyond the threshold level, ROS/RNS damage membrane lipids, proteins, or DNA, thus leading to biological damage. Out of several ROS-generating systems, macrophage stimulation in the alveolus and mitochondrial degeneration induced the overproduction of ROS in mitochondria via either the electron transport chain or cytochrome P450 as a metabolic by-product. The mitochondrial respiratory chain is primarily responsible for the overexpression of ROS, which, in turn, produces potential oxidants such as superoxide, H_2_O_2_, and other reactive species. H_2_O_2_ causes the expression of pro-inflammatory cytokines, such as IL-6, IL-1β, and TNFα, as well as iNOS, through a redox-sensitive transcription factor, NF-κB [32]. In the present study, qRT-PCR data showed that the transcriptional expressions of IL-1β and IL-18 were significantly high in response to the S protein. A similar trend was reflected in ELISA where the circulating level of IL-18 was found to be very high in the S protein-treated group. However, IL-1β in mice serum was below the detectable range during ELISA, probably because of its extremely short half-life in the circulatory fluid [33]. So, it may be hypothesized that NF-κB acted as a transcription factor for synthesizing IL-1β and IL-18. In response to the S protein, mRNA levels of inflammatory cytokines like IL-6, TNF-α, etc., in the lungs of BALB/c mice substantially increased. IL-10 played a paradoxical role in this study. The mRNA expression of IL-10 was upregulated with S protein administration. It was found further upregulated upon the therapeutic administration of UDE in both male and female mice, which probably suggests that it played an anti-inflammatory role in curbing the inflammatory response, unlike IL-6 and other pro-inflammatory cytokines.

According to studies, the S protein of SARS-CoV-2 interacts with the host either through ACE2 receptors with the help of TMPRSS2 or by other mediators like TLR-4, or TLR-2, and cellular stress is induced [34]. It further activates macrophages and neutrophils, elevates IL-6, IL-10, and other inflammatory markers, and contributes to the development of acute pulmonary inflammation [35].

UDE was able to bring down the transcriptional levels of both IL-1β and IL-18 and the translational level of IL-18. MMP-9 is a downstream target of the NF-κB signaling pathway, which was also upregulated, implying ECM remodeling during S protein-mediated lung injury. So, increased NF-κB levels were closely linked to the expression of MMP-9 and the emergence of the cytokine storm during the progression of the disease [36]. Mounting evidence also points to a direct connection between the production of ROS and the expression of iNOS, and consequently, the activation of the cytosolic multiprotein complexes and sensors of the innate immune system called “inflammasome” and further downstream signaling [37]. In this study, RT-PCR, ELISA, and Western blot data confirmed the significant upregulation of iNOS and caspase-1 in both the transcriptional and translational levels in response to S protein. Caspase-1 serves an essential function in the initiation of inflammation by the efficient cleavage and maturation of cytokines, IL-1β and IL-18, from their inactive precursors, both of which play important roles in host defense and mediate the pathogenesis during COVID-19 infection. RT-PCR also confirmed the significant transcriptional upregulation of these two cytokines which further downregulated with the therapeutic administration of UDE.

The activation of inflammasome resulted in excessive levels of cytokine production, which is referred to as cytokine storm and triggers a multifaceted and aberrant uncontrolled response. It involved pro-inflammatory cytokines, notably IL-6, IL-1β, and TNF-α. These cytokines, in turn, activated more macrophages, neutrophils, endothelium cells, and other immune cells to produce more superoxide, peroxynitrite, and free radicals which are harmful to mitochondria. We performed RT-PCR on all these cytokines, which appeared excessively upregulated in the S protein-treated group of mice. Interestingly, they all were downregulated in both male and female mice when UDE was administered orally.

Although the inflammasome and oxidative stress were both independently associated with COVID-19, it is unknown if these two mechanisms combine to increase disease severity. Here, we discovered a link between the elevated levels of SOD1 (through Western blot) and lipid peroxidation (spectrophotometrically), two characteristics of the oxidative stress response, in lungs treated with S protein, which substantially linked with high caspase-1 activity and excessive ROS formation. The literature also showed that the antioxidant enzyme SOD1 regulates both ROS in the cytoplasm and caspase-1 activation [38]. In this study, Western blot, as shown in Figure 8, depicted significant upregulation in SOD1 in S protein-treated tissues but again downregulated when UDE was applied to mice. Apart from the cytokine maturation or formation of inflammasome complex, caspase-1 regulates multiple cellular functions like inflammatory response, regulation of protein cleavage, apoptosis, etc. [39].

Increased ROS formation also leads to the rupture of mitochondria and the release of mitochondrial DNA. The release of mitochondrial DNA facilitates inflammatory response via the activation of the inflammasome. The consequences of mitochondrial damage are decreased ATP supply and cell death. The overall mitochondrial damage includes loss of size, shape, and integrity. It also may result in lowered membrane potential formation of MPT pore formation, leading to swelling. Additionally, S protein inhibited antioxidant enzymes’ ability to defend the mitochondria. NADH oxidase and succinate dehydrogenase activities are present in the mitochondrial respiratory complex I and complex II, respectively. A higher generation of ROS is suggested by increased NADH oxidase activity. Cytochrome C release is linked to the impairedelectron transport mechanism in mitochondria which facilitates apoptosis. Reduced SDH activity in lung epithelial cells also indicates less proton accumulation, which dissipates the potential of the mitochondrial membrane and allows the release of cytochrome C into the cytosol, which is confirmed in Figure 10. Our study clearly showed that UDE potentially resisted the excess ROS formation, both in vitro and in vivo; thereby inhibiting mitochondrial degeneration, leaking cytochrome C, and further downstream apoptotic events through caspases.

In our study, we noticed a significant upregulation of IL-6, IL-10, IL-1β, TNFα, iNOS, MMP-9, NF-κB, and MPO in response to S protein, which are the contributors to acute inflammatory response. The histopathology analysis of S protein-treated lungs of mice showed a substantial accumulation of inflammatory cells, lesions such as edema, inflammation, and a change in alveolar septal thickness. An elevated level of free radicals accompanies the over production of cytochrome C, which exacerbates the tissue damage and alters the redox equilibrium of the lungs.

The intranasal administration of UDE substantially reduced the permeation of inflammatory cells in the lungs of BALB/c mice. UDE demonstrated its antioxidant capability by reducing lipid peroxidation, protein carbonylation, and MPO activity during the healing of lung inflammation. Additionally, the cytoprotective effects of UDE were linked to the downregulation of inflammatory cytokines and restoring the homeostasis of free radicals and antioxidants. UDE administration provided significant protection in retaining the shape and integrity of mitochondria by regulating the cytoplasmic release of cytochrome C, and excess ROS production. The prophylactically UDE-treated group showed almost no localization of cytochrome C compared with the control, which indicates mitochondrial regeneration. On the other hand, the overproduction of ROS can also activate pro-apoptotic Bcl-2 family proteins by releasing cytochrome C from damaged mitochondria. It is detected by APAF-1 and pro-apoptotic caspase-3 and -9 [40,41]. The degree to which the apoptotic pathway is activated during COVID-19 has a direct association with the development of multiple complications. According to published research, the complex apoptosis process is influenced by caspases and the Bcl-2 protein family [42,43]. In the cascade of protease cleavage during apoptosis, the primary function of caspase-3 is to eliminate damaged cells. After activation, it cleaves proteins required for cell viability, thereby triggering apoptosis. When we tried to see the tissue damage in light of apoptosis, we found significant upregulations of caspase-3 and Bax, at both the transcriptional and translational levels, and the downregulation of Bcl-2 in the S protein-treated mice. RT-PCR data confirmed that therapeutic use of UDE reduced inflammation by normalizing the expression of those pro-apoptotic markers, increasing the expression of anti-apoptotic proteins like Bcl-2, scavenged ROS, and protecting the lung from oxidative damage. The terminal event of S protein-induced hyperinflammation is apoptosis, which results in cell lysis, the release of cytosolic contents to the extracellular space, along with tissue damage. In summary, the therapeutic administration of UDE was more effective against S protein-mediated damage. It probably inhibited hyperinflammation, mitochondriopathy, and apoptosis by controlling the expression of individual mediators of the signaling pathway, there by reducing the lung damage caused by the S protein. Nonetheless, UDE prevented the S protein mediated in many instances.

## 5. Conclusions

**Figure 11:**
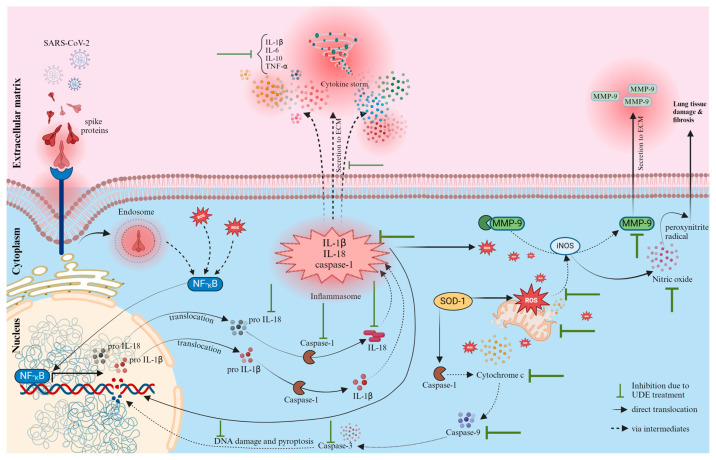
**Pathogenesis of COVID-19 in light of inflammasome activation, apoptosis, mitochondrial dysfunction, and oxidative stress.** The proposed model suggests that the binding of S protein of SARS-CoV-2 interacts with the ACE2 receptor which promotes endocytosis into the host cell. After entering, rapid viral replication causes a strong immune response, activation of inflammasome, and immune dysregulation, which is portrayed through various intermediates. Further downstream events include the leaking of inflammatory factors in the tissue space, producing excessive inflammation and cytokine storm. It may lead to immunopathological impairment and remodeling of the extracellular matrix by MMP-9, finally leading to severe pulmonary edema and pneumonia. It also causes mitochondrial dysfunction. This results in the exaggerated release of ROS, oxidative stress, and apoptosis. The hydroalcoholic formulation of Eupatorium mitigated the inflammatory response through the downregulation of caspase-1, IL-1β, and IL-18. It attenuates pro-inflammatory signals and cytokine release, thereby minimizing disease severity. UDE was also able to downregulate iNOS, MMP-9, cytochrome C, caspase-3, and caspase-9, helping to regenerate mitochondria and neutralize oxidative stress. The image was created using BioRender.com (www.biorender.com, accessed on 1 June 2024).

The current study showed that S protein treatment in both the cell culture and murine model resulted in a greater disruption of lung epithelial cell morphology, activation of the inflammasome, increased cell apoptosis, elevated pro-inflammatory cytokines, and extracellular matrix remodeling. S protein treatment also increased ROS production in the lungs and caused oxidative damage to cellular macromolecules, as well as mitochondrial dysfunction. Moreover, S protein treatment causes a significant influx of immune cells in the lungs; the upregulation of inflammatory mediators, including iNOS, IL-6, IL-1β, TNFα, cytochrome C, MPO, and MMP-9; and downstream mediators of inflammasome activation, caspase-1, and subsequently, IL-1β, IL-18, and pro-apoptotic factors, e.g., caspase-3, bax, in the systemic level, which played important roles in overall tissue damage and disease progression. In these comprehensive in vitro and in vivo studies, we explored the therapeutic potential of UDE in mitigating the oxidative stress in the lungs, and thus the hyperinflammation and immunopathology via the cytokine storm. We connected this mitochondrial damage to apoptosis, a pro-inflammatory form of cell death. To our knowledge, this is the first study documenting the mechanism by which UDE inhibits mitochondrial damage during hyperinflammatory response and oxidative stress, relevant to host immunity and several physiological and pathological conditions during the development of COVID-19 infection, which is outlined in Figure 11. To support the hypothesis put out in this work, additional pre-clinical research and clinical trialsare necessary.

## Figures and Tables

**Table 1:** **Schematic depicting the fold change in the proteins detected by ELISA in male and female mice separately.** ▲ denotes upregulation with respect to the control sets, and ▼ denotes downregulation with respect to the S protein-treated sets.

Proteins	Only S Protein (Fold Change w.r.t. Control)		UDE+ S Protein (Fold Change w.r.t. Only S Protein)		S Protein +UDE (Fold Change w.r.t. Only S Protein)	
	Male	Female	Male	Female	Male	Female
Caspase-1	22 ▲	17 ▲	6 ▼	2 ▼	8 ▼	8 ▼
iNOS	9 ▲	5 ▲	3 ▼	1.7 ▼	3.3 ▼	1.7 ▼
IL-18	9.4 ▲	8 ▲	3 ▼	3.6 ▼	13.3 ▼	10.5 ▼
IL-6	12 ▲	13.3 ▲	3.6 ▼	4 ▼	9 ▼	5.3 ▼

**Table 2:** Antioxidant and anti-inflammatory role of preventive and curative administration of UDE on S protein-induced damage of lung tissues in BALB/c mice. UDE was given prophylactically and therapeutically to mice before and after S protein administration. Lung tissue extracts from different groups of mice were used to assess MPO activity, total thiol group, and lipid peroxidation. From the mitochondrial fraction of the lung tissue extracts, activities of SDH, NADH oxidase, and ROS generation were assessed. The mean ± S.E. were used to represent the results. * indicates statistically significant with respect to control at *p* < 0.05, # denotes significant difference from S protein-treated group at *p* < 0.05 and ‘^’ denotes significance with respect to S protein-treated group at *p* < 0.05 in therapeutically UDE-treated group.

Animal Groups	Lipid Peroxidation Expressed as µmol Conjugated Diene/mg Protein		Myeloperoxidase Activity (Units/mg Protein)		Total Thiol Group (mM/mg Protein)		SDH (µm DCIP Reduced/mg Protein)		NADH Oxidase Expressed as Nmole Oxidase/min/mg Protein		ROS (DCF Intensity Offlourescence)	
	Male	Female	Male	Female	Male	Female	Male	Female	Male	Female	Male	Female
Control	1.52 ± 0.022	1.02 ± 0.632	0.56 ± 0.03	0.29 ± 0.01	142 ± 8.56	161.23 ± 10.23	2.66 ± 0.04	3.45 ± 0.23	1.23 ± 0.085	0.84 ± 0.02	62 ± 2.8	40 ± 3.1
S protein-treated	16.02 * ± 0.09	13.42 * ± 0.86	9.42 * ± 0.65	11.23 * ± 0.65	91 * ± 6.44	66.32 * ± 7.21	0.76 * ± 0.05	0.63 * ± 0.04	5.85 * ± 0.22	4.97 * ± 0.9	366 * ± 9.06	306 * ± 12.02
UDE+ S protein-treated	9.41 ^ ± 0.12	4.12 ^ ± 0.075	7.58 ^ ± 0.34	8.21 ^ ± 0.91	119 ^ ± 12.01	101.02 ^ ± 3.24	1.58 ^ ± 0.042	1.94 ^ ± 0.08	2.11 ^ ± 0.16	1.91 ^ ± 0.34	205 ^ ± 4.93	165 ^ ± 9.2
S protein + UDE treated	4.15 ^#^ ± 0.53	2.9 ^#^ ± 0.13	2.46 ^#^ ± 0.15	2.2 ^#^ ± 0.31	132 ^#^ ± 8.09	145.32 ^#^ ± 12.05	2.47 ^#^ ± 0.064	2.65 ^#^ ± 0.06	1.76 ^#^ ± 0.028	1.02 ^#^ ± 0.54	124 ^#^ ± 7.26	75 ^#^ ± 5.8

**Table 3:** **Tabular representation, depicting the fold change inthe proteins detected by Western blot in different groups of mice.** ▲ denotes upregulation with respect to the control sets, and ▼ denotes downregulation with respect to the S protein-treated sets.

Proteins	Only S Protein (Fold Change w.r.t. Control)	UDE+ S Protein (Fold Change w.r.t. Only S Protein)	S Protein +UDE (Fold Change w.r.t. Only S Protein)
SOD1	2.9 ▼	4.4 ▲	5.4 ▲
iNOS	2.8 ▲	3.4 ▼	3.8 ▼
Bcl2	2.4 ▲	1.2▲	1.6▲
Bax	2.2▲	2.3▼	3.3▼
Caspase-9	2.2 ▲	1.4 ▼	1.6 ▼

**Table 4:** Histological-scoring-related lung inflammation.

	Parameters →	Degree of Congestion	Extent of Hemorrhage	Severity of Inflammatory Cell Infiltrate	Proportion of Airspace Area in Percentage	Alveolar Diameter in µm (Mean ± SD)
Groups ↓						
Control		0/5	0/5	-	90%	12.84 ± 2.86
S protein-treated		5/5	5/5	+++	50%	25.2 ± 6.67
UDE + S protein-treated		2/5	1/5	+	80%	24 ± 7.05
S protein + UDE-treated		0/5	0/5	+	82%	17.56 ± 4.24

Note: The comparable microscopic fields of the lungs of six mice were examined, and the extent of lung damage was assessed using the criteria (modified) listed above [27,28]. The severity of the inflammation was judged per neutrophil infiltration and graded on a scale from nil to severe or—(nil), + (moderate) to +++ (severe). The airspace percentage denoted the critical ratio of the airspace area with the total area of one field under 100× magnification. Alveolar diameters were taken randomly for 10 intact alveoli in the microscopic field using Image J software. Merged, torn, and excessively large alveoli were excluded from measurement.

## Data Availability

No published data were used for the research described in this article.

## Supplementary Materials

The following supporting information can be downloaded at: https://www.mdpi.com/article/10.3390/diseases13020036/s1, Figure S1: Standardization of dose and exposure time for treatment of S protein and UDE on RAW 264.7 cells. Cell viability was assessed by MTT assay with different concentrations of S protein from 0.5% to 3% and UDE from 0.5% to 4% for varied time points. RAW 264.7 cells were maintained in culture media. (A) The cell viability decreased by 50% after 6 h when more than 1% concentration of S protein was applied in the media. (B) Viability of cells did not get affected significantly when the cells were treated with up to 4%UDE for 24 h; Figure S2: RAW 264.7 cells stained with EtBr-AO cocktail. The cells revealed the apoptotic states on exposure with S protein, pre-treatment with UDE followed by S protein and S protein treatment followed by UDE.

## Abbreviations

ACE2	Angiotensin-converting enzyme 2
ARDS	Acute respiratory distress syndrome
COVID-19	Coronavirus disease of 2019
ECM	Extracellular matrix
ELISA	Enzyme-linked immunosorbent assay
IL	Interleukin
iNOS	Inducible nitric oxide synthase
MMP	Matrix metalloproteinase
MPO	Myeloperoxidase
NF-κB	Nuclear factor kappa B
qRT-PCR	Real-time quantitative reverse transcription PCR
ROS	Reactive oxygen species
SARS-CoV-2	Severe acute respiratory syndromecoronavirus-2
SOD	Superoxide dismutase
TNFα	Tumor necrosis factor alpha
UDE	Ultradiluted hydroalcoholic formulation of *Eupatorium perfoliatum*
