# Supplementary material for: Ultradiluted Eupatorium perfoliatum Prevents and Alleviates SARS-CoV-2 Spike Protein-Induced Lung Pathogenesis by Regulating Inflammatory Response and Apoptosis
Source: Diseases. 2025 Jan 30;13(2):36. doi: 10.3390/diseases13020036 (PMC11854276; doi:10.3390/diseases13020036)
Supplement: Supplementary file 1 [file diseases-13-00036-s001.zip › Supplementary File S1.pdf]

A concentration of 1% up to 6 h and 4% up to 24 h were the standardized doses and durations of S protein and UDE respectively for cell culture experiments done throughout the study.

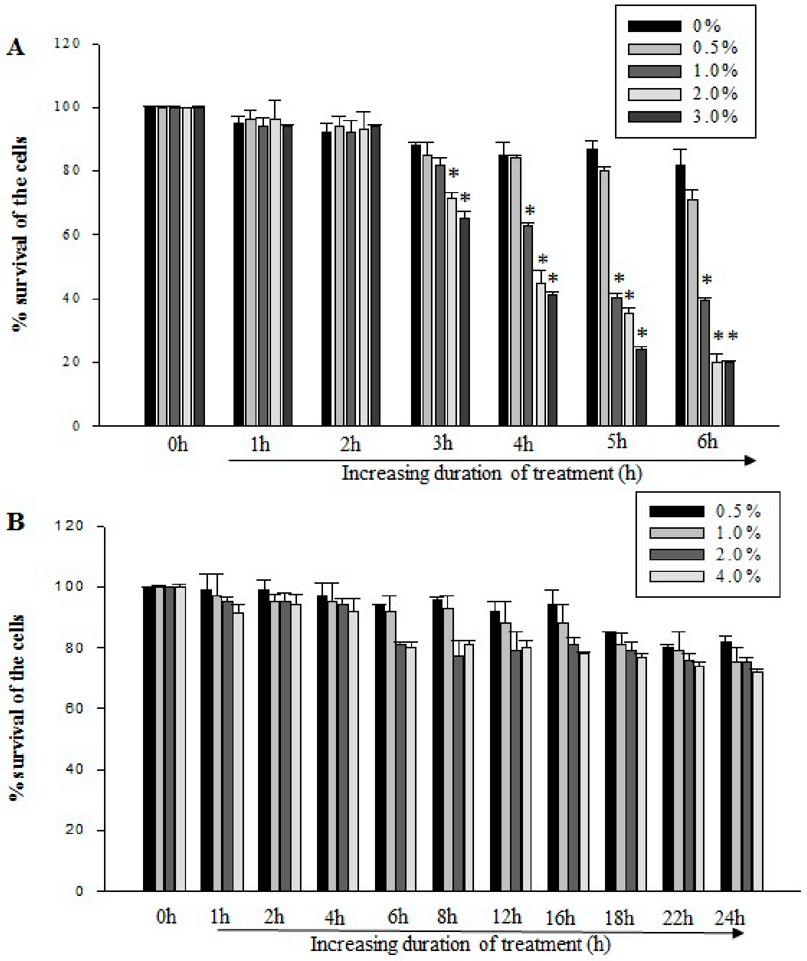

Supplementary Figure S1. **Standardization of dose and exposure time for treatment of S protein and UDE on RAW 264.7 cells** Cell viability was assessed by MTT assay with differentconcentrations of S protein from 0.5% to 3% and UDE from 0.5% to 4% for varied time points. RAW 264.7 cellswere maintained in culture media. (A) The cell viability decreased by 50% after 6 h when more than 1% concentration of S protein was applied in the media.(B) Viability of cells did not get affected significantly when the cells were treated withupto 4%UDE for 24 h.
