# Supplementary material for: Ultradiluted Eupatorium perfoliatum Prevents and Alleviates SARS-CoV-2 Spike Protein-Induced Lung Pathogenesis by Regulating Inflammatory Response and Apoptosis
Source: Diseases. 2025 Jan 30;13(2):36. doi: 10.3390/diseases13020036 (PMC11854276; doi:10.3390/diseases13020036)
Supplement: Supplementary file 1 [file diseases-13-00036-s001.zip › Supplementary File S2.pdf]

The EtBr-AO stained fluorescence micrographs of RAW 264.7 cells, depicted in Fig Suppl. 2, showed that almost 100% of the cells became yellow to orange which signified that cells became apoptotic to necrotic on S protein administration for 6h, whereas control cell nuclei appeared as uniformly brilliant green. UDE protected as well as healed the condition significantly by reducing the extent of apoptosis as the colour of nuclei were similar to the control cells. Further staining of apoptosis markers supported the finding came from this experiment.

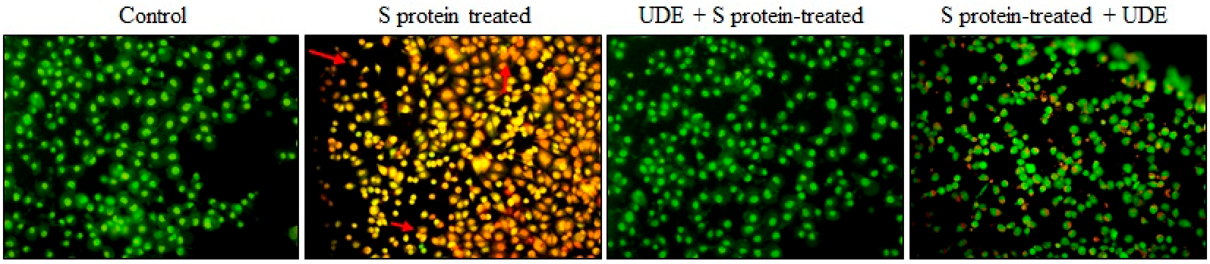

Supplementary Figure S2. **RAW 264.7 cells stained with EtBr-AO cocktail.** The cells revealed the apoptotic states on exposure with S protein, pre-treatment with UDE followed by S protein and S protein treatment followed by UDE.
